# Risk Scoring Systems for Preterm Birth and Their Performance: A Systematic Review

**DOI:** 10.3390/jcm12134360

**Published:** 2023-06-28

**Authors:** Amaro Ferreira, João Bernardes, Hernâni Gonçalves

**Affiliations:** 1 Faculty of Medicine, University of Porto, 4200-319 Porto, Portugal; amarof@sapo.pt; 2 Center for Health Technology and Services Research (CINTESIS@RISE), Faculty of Medicine, University of Porto, 4200-319 Porto, Portugal; jbernardes59@gmail.com; 3 Department of Obstetrics and Gynecology, Faculty of Medicine, University of Porto, 4200-319 Porto, Portugal; 4 Department of Obstetrics and Gynecology, Centro Hospitalar Universitário de São João, 4200-319 Porto, Portugal; 5 Department of Community Medicine, Information and Health Decision Sciences, Faculty of Medicine, University of Porto, 4200-319 Porto, Portugal

**Keywords:** preterm birth, risk scoring systems, performance, risk factors, fetal monitoring, pregnancy

## Abstract

*Introduction:* Nowadays, the risk stratification of preterm birth (PTB) and its prediction remain a challenge. Many risk factors associated with PTB have been identified, and risk scoring systems (RSSs) have been developed to face this challenge. The objectives of this systematic review were to identify RSSs for PTB, the variables they consist of, and their performance. *Materials and methods:* Two databases were searched, and two authors independently performed the screening and eligibility phases. Records studying an RSS, based on specified variables, with an evaluation of the predictive value for PTB, were considered eligible. Reference lists of eligible studies and review articles were also searched. Data from the included studies were extracted. *Results:* A total of 56 studies were included in this review. The most frequently incorporated variables in the RSS included in this review were maternal age, weight, history of smoking, history of previous PTB, and cervical length. The performance measures varied widely among the studies, with sensitivity ranging between 4.2% and 92.0% and area under the curve (AUC) between 0.59 and 0.95. *Conclusions:* Despite the recent technological and scientifical evolution with a better understanding of variables related to PTB and the definition of new ultrasonographic parameters and biomarkers associated with PTB, the RSS’s ability to predict PTB remains poor in most situations, thus compromising the integration of a single RSS in clinical practice. The development of new RSSs, the identification of new variables associated with PTB, and the elaboration of a large reference dataset might be a step forward to tackle the problem of PTB.

## 1. Introduction

According to the World Health Organization (WHO), a birth that occurs before 37 complete weeks of pregnancy is defined as a preterm birth (PTB). Based on gestational age, preterm births can be categorized as extreme preterm (less than 28 weeks), very preterm (28 to 32 weeks), or moderate to late preterm (32 to 37 weeks). Moreover, PTB can be classified into “spontaneous” (spontaneous onset of labor or following preterm premature rupture of membranes (PPROM)) and “indicated” (parturition initiated by the caregivers: induction of labor or elective cesarean for maternal or fetal indications or other non-medical indications) [1,2]. It is estimated that, annually, 15 million babies are born preterm, meaning that more than 1 in 10 babies are born too early [1].

PTB and its complications account for approximately 1 million child deaths each year, making it the leading cause of death in children under 5 years of age [3]. Current, cost-effective interventions could prevent three-quarters of these deaths [1,3]. Preterm-born surviving infants can have major health impairments due to the severe disruption in the normal developmental maturation of organ systems [4,5]. This immaturity of organ systems concomitant with higher levels of oxidative stress plays a role in the development of the preterm newborn main pathologies, examples of which are necrotizing enterocolitis, bronchopulmonary dysplasia, retinopathy of prematurity, intraventricular hemorrhage, and patent ductus arteriosus. Furthermore, preterm newborns are more susceptible to long-term neurodevelopmental impairments such as cerebral palsy, hearing and vision problems, and intellectual disability [6]. From a maternal health perspective, the experience of a PTB can impair the bonding to the baby, with less positive feelings, and lead to depression and anxiety postpartum [7]. In addition to its mortality and morbidity, PTB has also a significant financial impact not only on the families of preterm infants but also on health systems [8].

Certain factors represent potential risks for a spontaneous PTB. These risk factors can be classified as demographical, obstetrical, gynecological, and those related to the current pregnancy [9,10]. Demographical risk factors include maternal age (the higher the maternal age the greater the risk of a PTB [11]), ethnicity (black ethnicity is reported to have a higher risk of preterm birth compared to other ethnicities [12]), smoking, and the use of illicit drugs (increase the risk of PTB [13]), maternal stress, and other social factors (an association between low maternal education and PTB could be established [14]) [9,10]. Obstetrical and gynecological risk factors comprehend interpregnancy latency (increased odds of PTB were reported at less than 6 and 12 months interpregnancy intervals [15]), prior preterm delivery (PTB in a previous pregnancy is a strong risk factor for PTB in a subsequent pregnancy [16]), uterine, cervical and placental conditions (short cervical length, uterine anomalies, placental abruption, and placenta previa are associated with PTB [17,18,19,20]) [9,10]. Current pregnancy-related risk factors are uterine hemorrhage, fetal malformations (fetal malformations, in general, are associated with a greater risk of PTB [21]), multiple gestations (multiple gestations are a strong risk factor for both spontaneous and indicated PTB [22]), maternal and intra-amniotic infections (infectious conditions such as bacterial vaginosis, pyelonephritis, and chorioamnionitis have been linked to PTB [23,24,25]) [9,10].

Many studies have been developed with the purpose of creating a system by adding some of the risk factors mentioned together and evaluating its clinical significance concerning the prediction of PTB. Some other variables have been studied and incorporated in systems to predict PTB: fetal fibronectin found in cervicovaginal secretions [26]; maternal serum biomarkers, such as pregnant associated plasma protein-A (PAPP-A), human chorionic gonadotrophin (hCG), and alpha-fetoprotein (AFP) [27]; ultrasound markers [28]; and peripheral maternal blood microRNA [29].

Establishing risk factors for the prediction of PTB and understanding the relationship between certain (bio)markers and PTB can help identify women at risk allowing them to initiate adequate antenatal care and risk-specific treatment. In addition, studying all these PTB-related variables might give relevant information about possible causes of PTB and provide the opportunity to study particular interventions [10].

Reviews about risk scoring systems (RSSs) for the prediction of PTB have been published [30,31,32,33]. However, only one evaluated the performance of RSS for the prediction of PTB [32]. Accordingly, we conducted a systematic review to identify and compare RSSs developed to stratify the risk a pregnant woman with a PTB has, namely, regarding the variables considered in each RSS and the RSS predictive value of PTB, as well as the type of model used to develop the scoring system.

## 2. Materials and Methods

This systematic review was developed according to the PRISMA (Preferred Reporting Items for Systematic Reviews and Meta-Analyses) guidelines [34]. This review’s protocol was not documented or registered prospectively.

Studies were considered eligible if they studied or developed an RSS, based on specific variables, as to predict PTB and help to stratify the PTB risk, irrespective of the gestational age used as a threshold to define PTB. Studies that did not study or develop an RSS (uni or multivariable) that did not mention the variable(s) used in the RSS or that did not analyze the RSS predictive value were excluded. No restrictions were imposed concerning the studies’ participants and their pregnancy characteristics. Observational studies, for example, cohort, case-control, and cross-sectional studies were included. Editorials, clinical case reports, literature reviews, or incomplete publications (e.g., abstracts only) were excluded. Non-English and non-human published studies were also excluded.

Two databases were searched for studies: PubMed and Web of Science. We searched from inception to the 12 November 2022, the date on which we ran the final search. No time restrictions were imposed on the search. We screened the databases using the following search query: [(“preterm birth” OR “preterm delivery” OR “preterm labor” OR “preterm labour” OR “premature birth” OR “premature delivery” OR “premature labour” OR “premature labor”) AND (“risk” OR “risk factors”) AND (“scoring systems” OR “score*” OR “scoring algorithm”) AND (“validity” OR “validation” OR “assessment” OR “evaluation”)]. This query was built by adding together keywords considered pertinent for this review. Keywords adding no results were excluded from the query, such as “points system”. Search syntax was adapted for each database. No filters were applied to the searches.

The studies retrieved from the PubMed and Web of Science databases searches were exported to a reference manager (EndNote version 20), where duplicates were removed. Screening by title and abstract of the remaining studies was performed independently by two authors. Studies not meeting the inclusion criteria or not consistent with the purpose of this review were removed. Divergences between investigators were resolved by consensus.

After the screening phase, eligibility assessment was performed independently by the same two authors through the reading of the full-text articles, to certify their eligibility, using the inclusion and exclusion criteria defined. Divergences were also resolved by consensus. The reasons for the exclusion of studies both on screening and eligibility phases were registered.

Additionally, a manual search, of possible missing studies in the databases search, was performed in the list of references of the eligible studies, as well as in related review articles.

A structured data extraction form was developed to extract the data from the eligible studies. After testing it with some included studies, it was appropriately perfected. The data were independently extracted by two authors applying the previously developed data extraction form. Disagreements were discussed and solved by consensus. The following variables were collected: (1) study characteristics (year, country, study design, and sample size); (2) participant characteristics (exclusion and inclusion criteria); (3) outcome measure (PTB gestational age criteria considered, PTB type); and (4) scoring system characteristics (risk factors and variables considered, model used, model outcomes and output, performance analysis—reported by the area under the receiver operating characteristic curve (AUC), sensitivity, specificity, positive predictive value (PPV), and negative predictive value (NPV)—evaluation method and gestational age at RSS testing). Ambiguous or absent information was stated as “not reported”.

Methodological quality and study risk of bias were assessed based on the National Institutes of Health study quality assessment tool. This tool consists of a set of questions to evaluate a study’s internal validity and risk of bias. By applying this tool to a study, it is possible to classify them into one of three predefined categories: poor quality, fair quality, or good quality. Depending on the study design, different criteria were applied to assess the risk of bias in each study. Case-control and cohort studies were both assessed regarding the research question, study population, target population, sample size, and statistical analysis. In the case-control studies, it was also analyzed the inclusion/exclusion criteria, case and control definitions, selection of study participants, and exposure measurement. On the other hand, in the cohort studies, the timeframe was also analyzed to observe an effect, levels of exposure, and exposure and outcome measures and assessment. Intervention studies were assessed based on randomization, treatment allocation, blinding, the similarity of groups at baseline, dropout, adherence, outcome measures, power calculation, and intention-to-treat analysis. Study methodological quality was independently rated as good, fair, or poor by two authors. Disagreements were resolved by consensus.

## 3. Results

**Figure 1:**
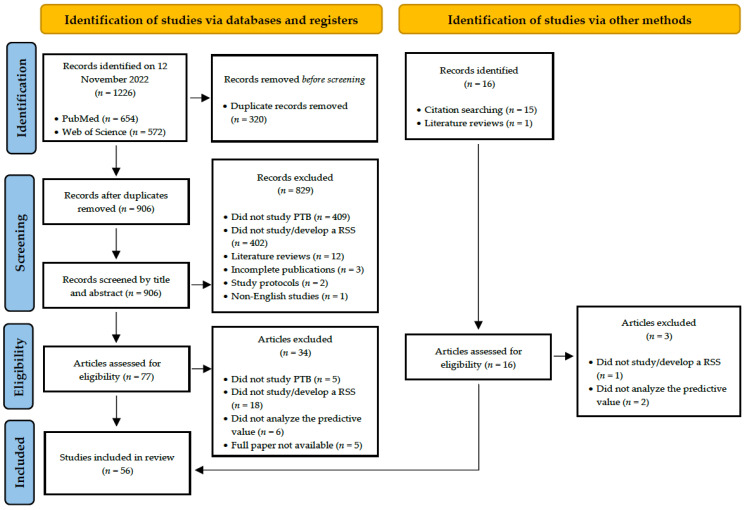
PRISMA flow diagram.

The final search of the two databases retrieved 1226 records. PubMed database search retrieved 654 records, whereas Web of Science database search retrieved 572 records. These records were extracted by a reference manager, where 320 duplicates were removed. From the 906 records screened by title and abstract, 829 did not proceed to the eligibility phase. The vast majority of the exclusions at this point were due to not studying PTB or not studying/developing an RSS (*n* = 409 and *n* = 402, respectively). Other reasons for article exclusion at this point were the study type (literature reviews, *n* = 12, and study protocols, *n* = 2), incomplete publications (*n* = 3), and non-English record (*n* = 1). After the screening, out of the 77 articles assessed for eligibility, 34 were excluded owing to not studying PTB (*n* = 5), not studying/developing an RSS (*n* = 18), not analyzing the RSS predictive power of PTB (*n* = 6), and unavailability of the full papers (*n* = 5). The unavailable papers were not available on the website of the respective journals. Literature reviews and reference lists from included articles were searched, and 16 records considered relevant to our review were identified. Of those 16, 3 articles were not eligible due to not studying/developing an RSS (*n* = 1) and not analyzing the RSS predictive value of PTB (*n* = 2). Therefore, in total, 56 studies [35,36,37,38,39,40,41,42,43,44,45,46,47,48,49,50,51,52,53,54,55,56,57,58,59,60,61,62,63,64,65,66,67,68,69,70,71,72,73,74,75,76,77,78,79,80,81,82,83,84,85,86,87,88,89,90] were included in this review. Figure 1 shows the PRISMA flow diagram, demonstrating the study selection process.

Regarding the study design of the studies included in this review, they were mainly observational cohort or case-control. A total of 46 cohort studies were included, and a total of nine case-control studies were included. Only one randomized controlled trial was included. In the study of methodological quality and risk of bias assessment, one study was rated as poor. A total of 28 studies were rated as good, and 27 were rated as fair. The most frequent reasons for studies not to be rated as good were the lack of adjustment for confounding variables, the non-definition of gestational age at the time of testing, and the poor definition of the study population.

The 56 studies included were conducted in a total of 20 different countries from 5 different continents. The countries with the most published studies under the eligibility criteria of this systematic review were the United States of America (USA, *n* = 20), the United Kingdom (UK, *n* = 6), and France (*n* = 5). Europe totals 25 included studies, considering studies from the UK, France, Germany, Italy, Sweden, Croatia, the Netherlands, Spain, Belgium, and Poland. Asia, South America, Oceania, and Africa were the least represented regions with four, two, two, and one published studies, respectively.

With respect to temporal analysis, only 14 studies were published until the year 2000, and among those, only three addressed an RSS that included laboratory variables in addition to variables obtained by clinical history and physical examination. A total of 42 studies included in this review were published in the 21st century, 16 of which were published in the last 5 years.

The study and the participant characteristics extracted from each of the 56 included studies, as well as the study of risk of bias assessment results, are outlined in Supporting Table S1. This table contains the following subset of characteristics defined in the data extraction form: year, country, study design, sample size, inclusion and exclusion criteria, and quality and risk of bias rating.

**Figure 2:**
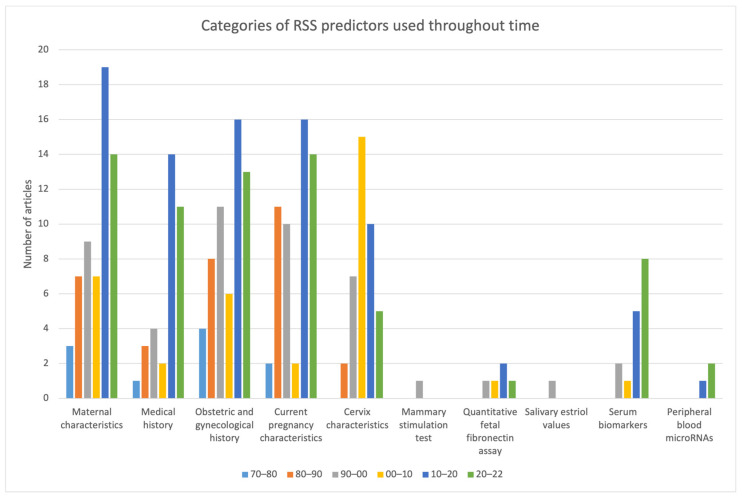
Distribution of RSS variables categories throughout time.

The most frequently incorporated variables in the RSS included in this review were maternal age, weight/BMI, history of smoking, history of previous PTB, and cervical length. In Figure 2, the variables are grouped in categories, and it is shown their distribution throughout the decades. Table 1 discriminates the variables that integrated the RSS addressed in the included studies. It also shows, by decade and in total, how many studies each variable was part of the RSS.

Not all studies included considered the same gestational age at PTB as the outcome. Prediction of PTB before 37 completed weeks of pregnancy was the most frequent outcome with approximately 69% of the studies included defining it as outcome. While some studies defined a single outcome, others did not restrict the outcome to the prediction of PTB before the completion of 37 weeks of pregnancy and studied the RSS prediction of PTB at different GA, such as before 32 (very PTB) and 34 completed weeks of pregnancy. Concerning the PTB classification as spontaneous or indicated, not all studies unanimously studied the same type of PTB. In total, 53% of the included studies focused exclusively on spontaneous PTB, whereas 22% focused on both spontaneous and medically indicated PTB.

RSSs addressed in the studies were built using different methods: univariate analysis (simple cutoff), multivariate models (linear and logistic regression) and, less frequently, machine learning models (artificial neural networks). Overall, considering all the included studies and the performance measures they presented, sensitivity ranged from 4.2% to 92.0%, specificity ranged from 41.5% to 99.3%, PPV ranged from 5.9% to 91.0%, NPV ranged from 69.2% to 100%, and AUC ranged from 0.59 to 0.95.

Table 2 gathers the eligible studies’ outcome measure, focusing on the PTB gestational age criteria considered and PTB type, and the scoring system characteristics, mainly the model used to build the RSS and its output and outcome, the risk factors and variables used, the performance analysis, reported by AUC, sensitivity, specificity, PPV and NPV, the evaluation method, and the gestational age at RSS testing.

## 4. Discussion

The present systematic review focused on the identification, characterization, and comparison of RSSs for the screening of the risk of PTB, with a focus on their performance. The extracted characteristics from each system included those related to study design and sample size; inclusion and exclusion criteria of participants; the predictors, considered model, outputs, and the performance; and the outcome measure and its applicability. To our knowledge, the only published systematic review addressing the performance of RSSs for PTB was published about 20 years ago [33].

The classical interpretation of an RSS may be assumed as a system including two or more predictors, based on which a sum of points produces a final score. However, we did not restrict our review to such interpretation, and thus, considered any system including one or more predictors, with different types of model output (also including probabilities).

The incorporation of clinical analysis results in RSS for PTB, according to the included studies in this review, started around the 90s decade. Since then, with the evolution of ultrasonography, the definition of new cervical ultrasonographic parameters and the discovery of biomarkers associated with PTB, the combined use of medical and obstetric history, maternal and pregnancy characteristics, ultrasonographic evaluation, and PTB biomarkers to develop an accurate RSS, capable of predicting PTB, has gradually increased.

Currently, attending to the fast technical development of ultrasonography, more accurate and reproducible ultrasound-based screening strategies can be performed for the prediction of PTB. Guidelines have been developed in order to provide recommendations and a consensus-based approach on this matter [91]. Although there has not been found a biomarker capable of accurately predicting PTB, many have been associated with it. These biomarkers associated with PTB are thought to be more predictive of PTB when used together in a model, instead of alone [27]. However, despite the undeniable progress in these areas and the development of RSSs combining all these components, their predictive value of PTB is still not as good as expected. The range of the RSS performance measures was wide, partly due to differences in inclusion/exclusion criteria of the studies’ participants and different RSS evaluation frameworks. In particular, the use of the whole sample validation scheme overestimates an RSS performance compared with cross-validation or internal/external validation. Therefore, it is difficult to clearly highlight an RSS for its predictive power and integrate it into the clinical practice.

The comparison between RSS should be carefully performed. With respect to the study design, the identified RSSs were tested in either cohort or case-control studies. The latter usually includes a higher prevalence of PTB in the sample than in the population, which may have, in some cases, led to an overestimation of the RSS predictive value, in terms of sensitivity and PPV. There were large differences in the inclusion and exclusion criteria between the reported studies. The identification of main groups of such criteria is advisable, such as singleton versus multiple pregnancies, the presence/absence of maternal and/or fetal pathologies, or obstetric history. The gestational age at PTB is considered as outcome and the gestational age at which the score was computed are also important factors, which influence the performance comparison between RSS. We did not report the existence of interventions in the course of pregnancy, as they are quite heterogeneous, but it may have a clear influence on the risk of PTB in the course of pregnancy.

One of the limitations of the presented review is related to the inherent possibility of any systematic review missing some relevant papers. However, in addition to the efforts put into the development of the search query, we did also a full search for other papers in the list of references of all papers in the eligibility phase, which led us to an increase of 30% in the papers initially identified in the screening phase. Another limitation might be the fact that we did not include RSSs, which combined PTB with other outcomes. Nevertheless, in our opinion, it does not provide a clear identification of risk factors strictly associated with PTB and avoids the comparison of their performance in terms of PTB.

## 5. Conclusions

This systematic review provides a characterization of most of the published RSSs for the assessment of the risk for PTB, which is, nowadays, not only one of the major causes of burden related to obstetrical care but also related to complications in the short and long term. This review suggests that the prediction of PTB and the risk stratification through RSSs is poor. Therefore, there is plenty of room for improvement in this field. Future studies should seek to develop new RSS, with good clinical applicability, based on PTB strongly associated variables, and should also seek to identify new variables, such as biomarkers or ultrasonographic parameters, related to PTB that can be combined with other variables to build better-performing RSSs. In order to account for the heterogeneity in PTB etiology and to provide an effective comparison between the available systems, a large reference dataset developed based on the joint efforts of different centers worldwide would be a step forward to tackle the problem of PTB.

## Figures and Tables

**Table 1:** Constituent variables of the RSS addressed in the included studies. Discrimination of each variable incorporation in RSS by decade and in total.

	76–80	80–90	90–00	00–10	10–20	20–22	Total
**Maternal characteristics:**							
Maternal age	1	1	2	2	5	4	15
Social class	1	-	1	-	1	1	4
Location of mother’s birth	-	-	-	-	1	-	1
Race/ethnicity	-	1	1	2	4	2	10
Marital status	-	2	1	1	-	-	4
Education grade	-	-	1	1	2	2	6
Height	-	1	1	-	2	1	5
Weight/Body mass index	1	2	2	1	4	4	14
**Medical history:**							
Preexisting diabetes	-	-	-	-	3	3	6
Preexisting hypertension	-	1	1	-	1	2	5
Smoking	1	2	3	2	5	2	15
Drug/alcohol abuse	-	-	-	-	3	3	6
Mental illness	-	-	-	-	2	1	3
**Obstetric and gynecological history:**							
Previous abortions	1	2	3	-	-	4	10
Previous preterm births	1	3	3	3	9	2	21
Previous livebirths < 2500 g	1	-	-	-	-	1	2
Previous large (4000 g+) infants	1	-	-	-	-	-	1
Previous cervical interventions	-	1	2	1	3	1	8
Parity	-	-	-	2	3	3	8
Interpregnancy interval	-	1	1	-	1	-	3
Uterine anomaly	-	1	2	-	-	2	5
**Current pregnancy characteristics:**							
Number of fetuses	-	2	2	-	3	4	11
Method of conception	-	-	-	-	2	2	4
Threatened abortion	1	-	-	-	-	-	1
Vaginal bleeding	1	1	2	-	2	1	7
Abruptio placentae	-	1	-	-	-	-	1
Placenta previa	-	2	1	-	-	1	4
Preeclampsia	-	1	-	-	-	1	2
Breech	-	1	-	-	-	-	1
Preterm premature rupture of membranes	-	1	-	-	2	1	4
Bacteriuria	-	1	3	-	1	2	7
Prenatal care visit initiation	-	-	-	1	3	2	6
Uterine contractions	-	-	1	-	1	-	2
Presence of membranes bulging into the vagina	-	-	-	-	1	-	1
Need and GA at transfer to a tertiary care center	-	-	-	-	1	-	1
Maternal weight gain per week	-	1	1	1	-	-	3
**Cervix characteristics:**							
Bishop score—clinical digital examination	-	1	3	2	2	-	8
Cervical assessment (cervical dilation—cervical length)	-	1	1	1	-	-	3
Cervical length	-	-	2	6	4	3	15
Cervical funneling	-	-	1		1		2
Mean gray value—Two-dimensional transvaginal ultrasound measurement of cervical length	-	-	-	1	-	-	1
Qualitative glandular cervical score	-	-	-	1	-	-	1
Endocervical glandular area	-	-	-	-	1	-	1
Anterior cervical angle	-	-	-	-	1	-	1
Elastography index	-	-	-	-	-	1	1
Strain pattern score	-	-	-	-	-	1	1
Blood velocity in the umbilical artery waveform	-	-	-	2	-	-	2
Blood velocity in the uterine artery waveform	-	-	-	2	1	-	3
**Mammary stimulation test**	-	-	1	-	-	-	1
**Quantitative fetal fibronectin assay**	-	-	1	1	2	1	5
**Salivary estriol values**	-	-	1	-	-	-	1
**Serum biomarkers:**							
Corticotropin-releasing hormone concentration.	-	-	1	-	-	-	1
Alpha-fetoprotein concentration	-	-	1	-	1	1	3
Human chorionic gonadotropin	-	-	-	1	-	1	2
Inhibin A	-	-	-	-	1	1	2
Total cholesterol	-	-	-	-	1	-	1
Insulin-like growth factor-binding protein	-	-	-	-	1	1	2
Sex hormone-binding globulin	-	-	-	-	1	1	2
Total bile acids	-	-	-	-	-	1	1
11-deoxycorticosterone	-	-	-	-	-	1	1
16-alpha hydroxyprogesterone	-	-	-	-	-	1	1
**Peripheral blood microRNAs**	-	-	-	-	1	2	3

**Table 2:** Outcome measure and RSS characteristics of each included study.

Article and Year	PTB GA	PTB Type	Model Used	Model Output and Outcomes	Variables Included	Performance Analysis	Evaluation Method	GA at Testing
1976 [35]	Not reported	Spontaneous	Product of relative risks	Low (<5) and high risk (≥5)	Maternal age, social class, weight, smoking, threatened abortion, previous abortions. Parous women only: previous PTB, previous livebirths < 2500 g, previous large (4000 g+) infants, previous antepartum hemorrhage	Multiparous: Sens. 25.3%; PPV 34.7%	Whole sample	Not reported
1980 [36]	<37 w	Spontaneous	Sum of points	Low (0–5), medium (6–9) and high risk (≥10)	Predictors related to socioeconomic status, medical history, daily habits, and current pregnancy	High/med. risk: Sens. 80% Spec. 72%; PPV 15%; NPV 98%	Whole sample	First prenatal visit, updated at 26–28 w
1984 [37]	<37 w	Not reported	Logistic regression	Low (<10%) and high risk (≥10%)	presence or absence of previous premature delivery, previous spontaneous abortion, abruptio placentae, placenta previa, severe preeclampsia, breech, smoking (no. cigarettes), PPROM, multiple pregnancy	Sens. 62.2%; Spec. 79.4%; PPV 22.7%	Whole sample	Not reported
1988 [38]	<37 w	Spontaneous	Cervical score (length minus dilatation)	Low (≥−1) and high risk (<−1)	Cervical assessment (subtracting dilatation from length)	PPV 76%	Whole sample	27–34 w
1989 [39]	<37 w	Both	Sum of points (based on Creasy [36])	Low (<10) and high risk (≥10)	Socioeconomic factors, previous medical history, daily habits and current pregnancy problems (18 variables)	Sens. 41.0%; PPV 24.6%; NPV 94.2%	Whole sample	First prenatal visit
1989 [40]	<37 w	Both	Logistic regression	Low (<1.83) and high risk (≥1.83)	pre-pregnancy weight <45.5 kg, black race, single marital status, history of PTB	Sens. 28.8%; Spec. 91.0%; PPV 21.9%	Whole sample	First prenatal visit, <28 w
1990 [41]	<37 w and <32 w (VPTB)	Spontaneous	Sum of points (based on Creasy [36])	Low (<10) and high risk (≥10)	Socioeconomic factors, previous medical history, daily habits and current pregnancy problems	Sens. 29%; Spec. 85%; PPV 16%; NPV 93%	Whole sample	First prenatal visit, <30 w
1991 [42]	<37 w and <34 w	Spontaneous	Cervical score (length minus dilatation)	For PTB < 34 w: Low (>0) and high risk (≤0)	Cervical assessment (subtracting dilatation from length)	Sens. 88%; Spec. 62%; PPV 75%; NPV 81%	Whole sample	<34 w
1994 [43]	<37 w	Spontaneous	Discriminant model	Low and high risk (no cutoff reported)	Positive mammary stimulation test result, soft cervix at 26 to 28 w, bacteriuria at the 1st prenatal visit, smoking during pregnancy, history of spontaneous abortion	Sens. 34.6%; Spec. 95.6%; PPV 47.4%, NPV 92.9%	Whole sample	26–28 w
1995 [44]	<37 w	Not reported	Sum of points (Creasy [36])	low (0–5), medium (6–9) and high risk (≥10)	Predictors related to socioeconomic status, past history, daily habits, and current pregnancy	Sens. 30.5%; Spec. 83.9%; PPV 44.3%; NPV 74.2%	Whole sample	First prenatal visit
1996 [45]	<37 w	Spontaneous	Logistic regression	Low (<20%) and high risk (≥20%)	Race, poor social environment, paying job during pregnancy, prior SPTD, acute or chronic lung disease, vaginal bleeding, contractions, BMI < 19.8, Bishop score	Multiparous: Sens. 24.2%; Spec. 92.1%; PPV 30.8%; NPV 89.4	Cross-validation (85% for training and 15% for testing)	23–24 w
1996 [46]	<37 w	Not reported	Simple cutoff	Low (<60 ng/mL) and high risk (≥60 ng/mL)	Fetal fibronectin assay of cervical and vaginal secretions, cervical length, presence of funneling, cervical index	Sens. 80.9%; Spec. 83.6%; PPV 79.2%; NPV 85.0%	Whole sample	24–36 w
1999 [47]	PTB within 72 h before 37 w	Spontaneous	Simple cutoff	Low (<2.1 ng/mL) and high risk (≥2.1 ng/mL)	Salivary estriol values	PPV 91%	Whole sample	≥21 w
1999 [48]	<37 w	Both	Based on likelihood ratios	Low and high risk (no cutoff reported)	Predictors related to socioeconomic status, past history, daily habits, and current pregnancy, corticotropin releasing-hormone and alpha-fetoprotein concentrations	Sens. 37%; Spec. 95%,	Whole sample	≥12 w
2003 [49]	<37 w	Both	Sum of scores	Numerical model (0–7) without specified categories	Blood velocity in the umbilical artery waveform (4 categories) and uterine artery blood flow velocity waveforms (5 categories)	AUC 0.71	Whole sample	Not reported
2003 [50]	<37 w	Spontaneous	Simple cutoff	Low (≤6.54) and high risk (>6.54)	Two-dimensional transvaginal ultrasound measurement of cervical length—Mean gray value	AUC 0.80; Sens. 82.1%; Spec. 72.5%; PPV 67.6%; NPV 85.3%	Whole sample	20–35 w
2004 [51]	<37 w and <35 w	Not reported	Simple cutoff	For PTB < 37 w: Low (<27) and high risk (≥27)	Human chorionic gonadotropin	For PTB < 37 w: Sens. 76%; Spec. 50%; PPV 85%; NPV 37%; Acc. 71%	Whole sample	25–35 w
2004 [52]	<32 w	Both	Simple cutoff	Low (>25 mm) and high risk (≤25 mm)	Cervical length	Sens. 75%; Spec. 90%; PPV 83%; NPV 81%	Whole sample	14–20 w
2005 [53]	<37 w	Spontaneous	Logistic regression	Low and high risk (threshold not reported)	CLEOPATRA I: cervical length and previous PTB; CLEOPATRA II: fetal fibronectin and previous PTB	CLEOPATRA I: AUC 0.69 CLEOPATRA II: AUC 0.81	Cross-validation (50% for training and 50% for testing)	24–35 w
2005 [54]	<34 w	Spontaneous	Simple cutoff	Cervical index: Low (<0.04) and high risk (≥0.04)	Ultrasound cervical assessment (cervical index and cervical score) and digital examination (Bishop score and cervical score)	Cervical index: AUC 0.85; Sens. 92%; Spec. 74%; PPV 26%; NPV 99%	Whole sample	27 w
2006 [55]	<37 w	Not reported	Simple cutoff	Low (<1.5) and high risk (≥1.5)	Umbilical artery pulsatility index	AUC 0.796	Whole sample	Not reported
2006 [56]	<34 w and 34–37 w	Spontaneous	Sum of the cervical mucus area and glandular invasion score	PTB 34–37 w: Low (>1) and high risk (≤1)	Cervical length and qualitative glandular cervical score	PTB 34–37 w: Sens. 50%; Spec. 96%; PPV 28%; NPV 98%	Whole sample	16–23 w
2006 [57]	<37 w	Not reported	Simple cutoff	Low (>24 mm) and high risk (≤24 mm)	Cervical length	Sens. 57.1%; Spec. 98.4%; PPV 66.7%; NPV 97.7%	Whole sample	16–23 w
2006 [58]	<37 w and <32 w	Both	Logistic regression	Low and high risk (no cutoff reported)	Maternal age, ethnicity, BMI, smoking status, obstetric history, previous cervical surgery, cervical length	For PTB < 37 w: AUC 0.667	Whole sample	22–25 w
2007 [59]	<32 w	Spontaneous	Logistic regression	Low and high risk (singletons > 0.04; twins > 0.10; triplets > 0.34)	Maternal age, maternal race, maternal education, marital status, parity, prenatal care visit initiation, maternal smoking, maternal weight gain per week, medical complications	Singletons: AUC 0.73; Sens. 24.6%; Spec. 93.5%; PPV 5.9%; NPV 98.7%;	Holdout (80% training and 20% testing)	Not reported
2008 [60]	<28 w, 28–30 w, 31–33 w, 34–36 w	Spontaneous	Logistic regression	Low and high risk (no cutoff reported)	Cervical length, obstetric history (parity and GA of previous delivery)	<28: AUC 0.92 28–30: AUC 0.84 31–33: AUC 0.82 34–36: AUC 0.65	Holdout	20–25 w
2011 [61]	<32 w	Not reported	Sum of points	Low and high risk (no cutoff reported)	PPROM, sonographic cervical length, gestational age at transfer, uterine contractions requiring tocolysis, multiple pregnancies, and vaginal bleeding	Training: AUC 0.79 Validation: AUC 0.72	Holdout (737 training and 169 validation)	22–32 w
2011 [62]	<34 w	Spontaneous	Logistic regression	Low and high risk (no cutoff reported)	Maternal age, height, racial origin, smoking status, method of conception and obstetric history	AUC 0.668; Sens. (FPR 10) 27.5%	Whole sample	11–14 w
2012 [63]	<37 w	Not reported	Logistic regression	Low (<2) and high risk (≥2)	Initial cervical dilation, obstetric history (parity and previous PTB), tobacco use	AUC 0.73; Sens. 79%; Spec. 50%; PPV 46%; NPV 82%	Internal validation with bootstrapping	22–34 w
2012 [64]	<32 w	Not reported	Logistic regression	Low and high risk (no cutoff reported)	Cervical dilation, obstetric history, presence of membranes bulging into the vagina and infection	AUC 0.88	Whole sample	15–24 w
2012 [65]	<37 w	Spontaneous	Logistic regression	Low (<0.1) and high risk (≥0.1)	Maternal characteristics (maternal age, maternal ethnicity, socioeconomic status, living in a deprived area), obstetric history (parity, pre-existent diabetes mellitus, previous PTB, history of cervical surgery, psychiatric disorder, drug abuse), current pregnancy (booking visit ≥18 w of gestation, vaginal bleeding <20 w of gestation, male fetal sex)	Sens. 4.2%; Spec. 99.3%; PPV 19.4%; NPV 96.3%	Internal validation with bootstrapping	Around 20 w
2013 [66]	<37 w	Spontaneous	Combined cutoff and categoric variable	High risk for short cervix (<20 mm) + echogenicity	Cervical length and endocervical glandular area	Sens. 34.4%; Spec. 41.5%; PPV 64.7%; NPV 77.8%	Whole sample	24–34 w
2013 [67]	<37 w	Spontaneous	Logistic regression	Low and high risk (no cutoff reported)	Maternal age, body mass index, smoking status, history of late miscarriage and/or preterm delivery, and previous delivery to term	AUC 0.618; Sens (FPR 10) 23.3% PPV 7.4%; NPV 97.2%	External validation	1st trimester
2013 [68]	<37 w	Both	Logistic regression	Low and high risk (no cutoff reported)	Maternal characteristics (maternal degree, prepregancy diabetes, previous PTB, previous live birth, and maternal BMI), routine serum analytes (AFP and inhibin A), cholesterol (first-trimester TC and TC change between trimesters [second TC trimester—first trimester TC])	AUC 0.70; Sens.31.2%; Spec. 90.6%; PPV 21.3%; NPV 94.2%	Whole sample	1st and 2nd trimesters
2015 [69]	<37 w, <34 w, <30 w	Spontaneous	Simple cutoff	Low (<200 ng/mL) and high risk (≥200 ng/mL)	Quantitative fetal fibronectin	<30: AUC 0.82 <34: AUC 0.74 <37: AUC 0.67	Whole sample	18–28 w
2016 [70]	<34 w	“Indicated”	Simple cutoff	Low and high risk (cutoff corresponding to 85% spec.)	Uterine artery pulsatility index	AUC 0.93; Sens. 87%	Whole sample	24–34 w
2016 [71]	<37 w	Spontaneous	Simple cutoff	Low (>−1.37) and high risk (≤−1.37)	Insulin-like growth factor-binding protein and sex hormone-binding globulin	AUC 0.75; Sens. 75%; Spec. 0.74%	Holdout (discovery, verification and validation subsets)	17–29 w
2016 [72]	<37 w	Spontaneous	Parametric survival model	High risk for individual probability > 10%	Cervicovaginal fluid quantitative fetal fibronectin, cervical length, previous PTB or PPROM	AUC 0.77; Sens. 74.5%; Spec. 63.5%; PPV 26.5%; NPV 93.4%.	Holdout (50% training and 50% validation)	22–30 w
2017 [73]	<34 w	Spontaneous	Logistic regression	Low and high risk (no cutoff reported)	Anterior cervical angle, cervical length and maternal characteristics (maternal age and previous history of PTB)	Sens. 37.6%; Spec. 90%	Whole sample	20–25 w
2017 [74]	<34 w, 34–38 w	Spontaneous	Sum of categorical variables	Low and high risk (no cutoff reported)	Peripheral blood mononuclear cell microRNA (miR-148a, -301a, -671, -181a, -210, -1267, -223, and -340)	PTB 34–38 w: AUC 0.92; Sens. 86%; Spec. 84%	Holdout (50% training and 50% validation)	4–13 w
2018 [75]	<37 w	Spontaneous	Logistic regression	Low and high risk (no cutoff reported)	Race or ethnicity, age at delivery, education, payment for prenatal care, parity, location of mother’s birth, body mass index, preexisting diabetes, preexisting hypertension, reported smoking, reported drug/alcohol abuse, mental illness, sickle cell anemia, previous cesarean delivery, previous PTB, interpregnancy interval	AUC 0.591	Holdout (2/3 training and 1/3 testing)	1st trimester
2018 [76]	<37 w	Spontaneous	Simple cutoff (Cervical texture based score)	Low (>−0.68) and high risk (≤−0.68)	Cervical texture features	Sens. 70.4%; Spec. 77.4%	Leave-one-out cross validation	19–25 w
2018 [77]	<33 w	Not reported	Simple binary model	Low (no funneling) and high risk (funneling)	Cervical funneling	Sens. 51%; Spec. 61%	Whole sample	10–28 w
2019 [78]	<37 w within 48 h and 7 days	Not reported	Logistic regression	High-risk for probability > 0.5	Number of fetuses, age (mother), gravidity, parity, length (mother), weight (mother), BMI, gestational age at admission, duration ruptured membranes, method of conception, smoking history, alcohol usage, drug usage, history of cesarean section, race (mother), and admission indications	Within 7 days: AUC 0.83; Acc. 80%; Sens. 60%; Spec. 90%	5-fold cross validation	24–37 w
2020 [79]	<37 w	Spontaneous	Logistic regression	High risk for cervical length > 41.1 mm, elastography index > 1.325 and strain pattern = 2	Elastography index, Strain pattern score, Cervical length	AUC 0.90; Sens. 52%; Spec. 96%	Whole sample	20–34 w
2020 [80]	<37 w	Both	Simple cutoff	Low (<32.1 umol/L) and high risk (≥32.1 umol/L)	Total bile acids	AUC 0.62; Sens. 55.6%; Spec. 72.6%; PPV 59.5%; NPV 69.2%	Whole sample	Not reported
2020 [81]	<37 w	Not reported	Logistic regression	Low and high risk (no cutoff reported)	Age, family situation, health coverage, gestity, parity, scarred uterus, prenatal interview	Validation dataset: AUC 0.63	External validation (prospective validation dataset)	1st trimester
2020 [82]	<37 w	Spontaneous	Simple cutoff	Low (>25 mm) and high risk (≤25 mm)	Cervical length	Sens. 70%	Whole sample	25–35 w
2020 [83]	<32 w	Both	Simple cutoff	Low and high risk (no cutoff reported)	Insulin-like growth factor-binding protein 4 and sex hormone-binding globulin	AUC 0.71	Whole sample	17–22 w
2020 [84]	<35 w	Spontaneous	Sum of 12 dichotomized variables	Low (<2) and high risk (≥2)	Peripheral blood microRNA (miR-181a-3p, miR-221-3p, miR-33a-5p, miR-6752-3p, miR-1244, miR-148a-3p, miR-1-3p, miR-1267, miR-223-5p, miR-199b-5p, miR-133b and miR-144-3p)	AUC 0.80; Sens. 89%; Spec. 71%; PPV 23%; NPV 99%	Holdout (50% training and 50% validation)	6–13 w
2021 [85]	<32 w	Both	Logistic regression	Low and high risk (no cutoff reported)	Progesterone metabolites 11-deoxycorticosterone, 16-alpha hydroxyprogesterone, parity, age, race, BMI, prior preterm deliveries, prior miscarriages	AUC 0.94; Sens. 91%; Spec. 87%; PPV 63%; NPV 98%	Whole sample	Late 1st/Early 2nd trimester
2021 [86]	<37 w	Not reported	Relative risk weight converted into 0–100 score	For the 3rd trimester Low (<2) and high risk (≥2)	71 risk factors that constituted six groups: anatomical, behavioral, demographic, disease, historical, and environmental	For the 3rd trimester: AUC 0.73; Sens. 53.1%; Spec. 82.4%; PPV 16.8%; NPV 96.4%	Holdout (80% training and 20% test)	All pregnancy
2021 [87]	<34 w	Spontaneous	Sum of points	For 26–28 w: Low (<100) and high risk (≥100)	22–24 w: primiparity, monochorionicity, prepregnancy BMI, previous premature or late abortion, and cervical length. 26–28 w: primiparity, monochorionicity, history of premature or late abortion, cervical length and cervical length shortening rate.	AUC 0.88; Sens. 69.4%; Spec. 88.6%; PPV 63.2%; NPV 91.1%	Holdout (70% training and 30% validation)	22–24 w and 26–28 w
2021 [88]	<37 w	Both	Logistic regression and machine learning (artificial neural networks)	Low and high risk (no cutoff reported)	23 possible predictors in the 1st trimester, 35 possible predictors in the 2nd trimester	Artificial neural networks 2nd trimester AUC 0.80; Sens. 62.7%; Spec. 84.6%; PPV 23.2%; NPV 97.0%	Holdout (2/3 for training and 1/3 for validation)	1st and 2nd trimesters
2022 [89]	<37 w	Both	Least absolute shrinkage and selection operator via logistic regression	Levels 0, 1, 2, and 3 (lower to higher risk)	26 variables grouped as drugs, hospital diagnosis, inpatient procedures, exemptions, outpatient services, socio-demographic conditions, and use of assisted medical conception techniques	AUC 0.61	holdout (70% training and 30% testing)	Pre-pregnancy
2022 [90]	<37 w	Spontaneous	Linear combination	Low (<13.89) and high risk (≥13.89)	Maternal blood early B cell factor gene-based microRNA transcripts (MIR4266, MIR1251, MIR601 and MIR3612)	AUC 0.82; Sens. 81%; Spec. 72%	Cross-validation	27–33 w

Abbreviations: acc., accuracy; AFP, alpha fetoprotein; AUC, area under the receiving operator characteristic curve; BMI, body mass index; FPR, false positive rate; GA, gestational age; PPROM, preterm premature rupture of membranes; NPV, negative predictive value; PPV, positive predictive value; PTB, preterm birth; sens., sensitivity; spec., specificity; TC, total cholesterol; w, week(s).

## Data Availability

The data presented in this study are available in this article.

## Supplementary Materials

The following supporting information can be downloaded at: https://www.mdpi.com/article/10.3390/jcm12134360/s1, Table S1: Study and participants characteristics of each included study; quality and risk of bias ratings.
