# Supplementary material for: Risk Scoring Systems for Preterm Birth and Their Performance: A Systematic Review
Source: J Clin Med. 2023 Jun 28;12(13):4360. doi: 10.3390/jcm12134360 (PMC10342801; doi:10.3390/jcm12134360)
Supplement: Supplementary file 1 [file jcm-12-04360-s001.zip › jcm-2451432-supplementary.pdf]

Table S1. Study and participant characteristics of each included study; quality and risk of bias ratings.

Article and year	Country	Study design	Sample size (n)	Inclusion criteria	Exclusion criteria	Risk of bias
1976 [35]	UK	Case-control	793	Singleton deliveries	Not reported	Fair
1980 [36]	New Zealand	Cohort	966	Patients registering for delivery	Expected date of confinement less than 6 weeks from the date of the first prenatal visit	Good
1984 [37]	USA	Case-control	2865	Pregnant women	Not reported	Poor
1988 [38]	Zimbabwe	Cohort	223	Twin pregnancy	Primigravida; induced labor and pre-labor cesarean section; delivery <28 weeks of GA; unknown GA; cervical cerclage; low-lying placenta precluding vaginal examination.	Fair
1989 [39]	USA	Cohort	7329	Patients registering for prenatal care	Not reported	Fair
1989 [40]	USA	Cohort	4591	Patients registering for prenatal care	Not reported	Fair
1990 [41]	USA	Cohort with intervention	7478	Women who registered for obstetric care	Fetal anomaly	Fair
1991 [42]	USA	Cohort	93	Multifetal gestations	Not reported	Fair
1994 [43]	USA	Cohort	265	Nulliparous with singleton pregnancies	Placenta previa or PPROM	Fair
1995 [44]	USA	Cohort with intervention	433	Publicly served pregnant women	Spontaneous abortion, multiple births, ethnic group with too few records	Fair
1996 [45]	USA	Cohort	2929	Pregnant women with singleton gestations with obstetric sonogram	Fetal death, major life-threatening congenital fetal malformations, or placenta previa	Good
1996 [46]	Italy	Cohort	108	Singleton gestation; GA between 24 and 36 weeks; cervical dilatation <3 cm; intact amniotic membranes; absence of other maternal or fetal complications at admission and during pregnancy; cervical and vaginal secretion sampling and ultrasonographic assessment of the cervix obtained at admission	Not reported	Fair

1999 [47]	USA	Cohort	601	Singleton pregnancy; pregnancy between 21-25 weeks	Symptoms of PTL, tocolytic therapy, placenta previa, cervical changes necessitating cerclage, ruptured fetal membranes, preeclampsia, medications known to affect hormone levels, planned cesarean delivery, major congenital fetal abnormalities, IUGR, fetal death, maternal medical complications	Good
1999 [48]	Australia	Cohort	819	Pregnant women	Uncertain GA, treatment with systemic corticosteroids	Fair
2003 [49]	Sweden	Cohort	633	Pregnant women referred for Doppler examination as part of perinatal surveillance in high-risk pregnancies	Not reported	Fair
2003 [50]	Germany	Cohort	68	Pregnant women	Multiple gestation, cerclage, placenta previa, major fetal anomaly	Good
2004 [51]	Turkey	Cohort	102	Diagnosis of threatened labor with intact membranes, singleton pregnancy, no vaginal bleeding, GA between 25-35 weeks	Placenta previa, abruptio placenta, fetal anomaly, PPROM, hypertension, non-reassuring fetal status	Fair
2004 [52]	Israel	Cohort	36	Triplet pregnancy	Multifetal pregnancy reduction, indicated induction of labor before the onset of spontaneous delivery	Good
2005 [53]	Germany	Cohort	170	Singleton pregnancies with threatened PTL between 24-35 weeks	Pregnancies complicated by cervical cerclage, cervical dilation ≥ 3cm, placenta previa, clinical criteria of intrauterine infection, vaginal bleeding of unknown origin, fetal growth restriction, pre-eclampsia, suspected fetal asphyxia or major fetal anomaly	Good
2005 [54]	France	Cohort	328	Twin pregnancies with routine ultrasound examinations scheduled between 21 and 23 completed weeks of gestation or between 26 and 28 weeks	Prophylactic cerclage, placenta previa, major fetal anomaly, twin-twin transfusion syndrome, ruptured membranes, or undetermined GA	Good
2006 [55]	Sweden	Cohort	217	Singleton pregnancies admitted for Doppler ultrasound surveillance due to suspected IUGR	Not reported	Fair
2006 [56]	Croatia	Cohort	327	Nulliparous women with uncomplicated singleton pregnancy	Suspected infection, cervical and vaginal infection on swabs, history of cervical surgery, developmental malformations of the Müllerian ducts, cervical cerclage before enrollment, contractions, medical conditions that are risk factors for PTB, major congenital fetal anomalies, intrauterine fetal death	Fair

2006 [57]	Croatia	Interventional	282	Asymptomatic, nulliparous women with uncomplicated singleton pregnancy	Suspected infection, cervical and vaginal infection on swabs, history of surgical procedure on the cervix, developmental malformations of the Müllerian ducts, cervical cerclage before enrollment, medical conditions that are risk factors for PTB, major congenital fetal anomalies, intrauterine fetal death	Fair
2006 [58]	UK	Cohort	39284	Women with singleton pregnancies attending for routine antenatal care	Women with major fetal abnormalities, painful regular uterine contractions, or a history of ruptured membranes or cervical cerclage in-situ	Good
2007 [59]	USA	Cohort	298844 (singletons), 104536 (twins), 3868 (triplets)	Singleton, twin, and triplet pregnancies	Stillbirths, births with no information of GA, absence of tobacco use information	Fair
2008 [60]	UK	Cohort	58807	Women with singleton pregnancies undergoing routine antenatal care	Major fetal abnormalities, painful regular uterine contractions, or history of ruptured membranes or cervical cerclage in situ	Good
2011 [61]	France	Cohort	906	Women transferred to a tertiary care center for threatened preterm delivery	Pregnancies complicated by PPROM without uterine activity or cervical changes, preeclampsia, fetal growth restriction, twin-to-twin transfusion syndrome, in utero fetal death, or major fetal anomaly	Good
2011 [62]	UK	Cohort	33370	Women attending their routine first hospital visit in pregnancy between week 11 and 13 weeks and 6 days gestation, singleton pregnancy, delivering a phenotypically normal neonate at or after 24 weeks gestation	Major fetal abnormalities, those ending in termination, miscarriage or fetal death before 24 weeks and iatrogenic delivery before 34 weeks	Good
2012 [63]	USA	Cohort	583	Singleton pregnancies, diagnosed PTL	Multiple gestations, major fetal anomaly, intrauterine fetal demise, severe preeclampsia before enrollment, chronic steroid or immunosuppressive drug use, active immunologic disease, acute systemic febrile illness, pregestational diabetes	Good
2012 [64]	France	Cohort	85	Singleton pregnancy receiving emergency cervical cerclage	Dilated cervix without visible amniotic sac, prophylactic cerclage earlier in the current pregnancy, PPROM, obstetric disorder, clinical signs of chorioamnionitis	Good

2012 [65]	The Netherlands	Cohort	1524058	Singleton pregnancies resulted in birth where fetus was alive	pregnancy ending before 22 weeks, unknown GA, birthweight <500g, multiple pregnancies, antepartum fetal mortality, maternal ethnicity unknown, preterm inductions of labor and preterm primary cesarian sections	Good
2013 [66]	Turkey	Cohort	85	Pregnant patients with threatened labor. Administered tocolytic therapy to all of them	Cervical dilatation revealing an established situation of PTL, urinary infection and/or any other comorbid conditions that pose potential risk to induce uterine contractions	Good
2013 [67]	France	Cohort	17341 + 2412	Singleton pregnancies, an antenatal consultation before 14 weeks, and a delivery after 24 weeks	Pregnancies with imprecise dates, fetal deaths, medical terminations of pregnancy and cases of induced preterm delivery before 37 weeks	Good
2013 [68]	USA	Cohort	2509	Women undergoing routine prenatal testing in the 1st and/or 2nd trimesters, infants born alive at a GA ≥ 20 weeks, women with serum analyte results collected in both the 1st and 2nd trimester	Multiple gestations, congenital anomaly, unknown birthweight, birthweight for GA > 3 SD from the mean, serious infection, second pregnancy, term births that were treated with either cerclage or tocolysis, aberrant cholesterol measures between trimesters	Good
2015 [69]	UK	Cohort	130	Asymptomatic women with twin pregnancies with valid index test (qfFN) between 18 and 28 weeks of gestation	Missing GA, threatened PTL, prior sexual intercourse (within 24 hours) or frank bleeding visible on the swab	Good
2016 [70]	USA	Cohort	314	Singleton pregnancy, ultrasound biometry below the 10th percentile for GA, first ultrasound for dating performed prior to 24 weeks of gestation, no evidence of preeclampsia at the ultrasonographic examination, venipuncture performed within 2 days of the ultrasonographic examination	PTL, PPROM, preeclampsia, or impaired fetal growth, known major fetal anomaly or fetal demise, active vaginal bleeding, serious medical illness (renal insufficiency, congestive heart disease, chronic respiratory insufficiency, or active hepatitis)	Good
2016 [71]	USA	Cohort	4509	Asymptomatic pregnant women	Multiple gestations, known or suspected major fetal anomalies	Good
2016 [72]	UK	Cohort	1249	High-risk asymptomatic women attending a preterm surveillance clinic	Blood-stained swab, sexual intercourse within the last 24 hours, multiple pregnancies, without cervical length measurement, with insufficient or absent qfFN	Good
2017 [73]	Chile	Case-control	318	Pregnant women with a transvaginal ultrasound	Incomplete delivery outcomes, without cervical image, aneuploidies, congenital malformations, multiple pregnancies, stillbirths	Fair

2017 [74]	USA	Case-control	39	Index cycle between September 2012 and September 2015, frozen Trizoltreated blood sample available from the first trimester, singleton delivery, spontaneous delivery, if delivery was preterm, delivered baby with no obvious birth defects	Post-term delivery (>42 weeks) and cases where the clinic failed to receive delivery details	Good
2018 [75]	USA	Cohort	2339696	Singleton births with gestations 22–42 weeks in a birth cohort	Birthweight outside of three SD from the mean by sex	Good
2018 [76]	Spain	Case-control	310	Women with a singleton pregnancy and an sPTB risk factor (history of sPTB or miscarriage ≤16 weeks, and cervical intervention or Mullerian malformation)	Receiving treatment to prevent sPTB, such as progesterone, cervical cerclage, cervical pessary, medical indicated PTB	Good
2018 [77]	Germany	Cohort	166	Singleton asymptomatic pregnancies with high-risk factors (eg recurrent miscarriage, previous PTB, recurrent vaginal bleeding, cervical surgical procedures, infections)	Stillbirth, fetal congenital anomalies, uterine anomalies, preeclampsia, and metabolic diseases	Fair
2019 [78]	Belgium	Cohort	1145	High-risk pregnancies occurring between 24 and 37 weeks of gestation	Not reported	Fair
2020 [79]	Germany	Case-control	335	Pregnant women between the 20th and 34th week of gestation	Maternal age < 18 years, GA < 20th and > 34th week, known causes for PTB such as multiple pregnancies, premature rupture of membranes, previous cervical surgery, PTL contractions, as well as maternal, fetal, or uterine pathologies that lead to iatrogenic PTB	Good
2020 [80]	Poland	Cohort	86	Intrahepatic cholestasis of pregnancy	Other conditions causing pruritus, chronic/acute liver and bile duct diseases, pre-eclampsia, HELLP syndrome	Fair
2020 [81]	France	Cohort	12983 + 6914	Singleton pregnancy	Multiple pregnancies	Fair
2020 [82]	Brazil	Cohort	95	Women with singleton pregnancies and intact membranes given a diagnosis of PTL and who underwent transvaginal evaluation	Multiple gestations, maternal comorbidities, history of conization of the cervix, infections, postnatal diagnosis of congenital anomalies, the presence of cervical dilation of ≥4 cm at admission, and the absence of follow-up	Good
2020 [83]	USA	Cohort	847	Age ≥18 years, singleton pregnancy, no symptoms of PTL or membrane rupture	Planned delivery before 37 weeks, major anomalies or chromosomal disorders, planned cerclage, or progesterone use after 14w	Fair

2020 [84]	USA	Case-control	157	Singleton delivery of a live born infant, maternal BMI <30 kg/m2, blood sample drawn between 6w and 12 weeks 6 days of gestation, neonate without detectable birth defects	Not reported	Fair
2021 [85]	USA	Cohort	58	Women enrolled in the biobank with a singleton gestation	Use of steroids or anticoagulants prior to the sample collection	Fair
2021 [86]	USA	Cohort	29166	Women within the data set of total identified pregnancies who could be matched to the delivery of a live infant	Women within the data set of total identified pregnancies whose pregnancy was lost or who could not be matched to an infant at birth	Fair
2021 [87]	China	Case-control	309	Twin pregnancies	Uncertain pregnancy date, maternal or fetal indications for iatrogenic PTB, twin birth weight < 500 g, GA at birth < 24 weeks, genetic or structural abnormalities of either fetus, stillbirth of one or two fetuses, monoamniotic or monochorionic twin pregnancy complicated by twin transfusion syndrome or twin anemia-polycythemia sequence, placement of cervical cerclage, incomplete maternal data, delivery at a medical center other than ours.	Good
2021 [88]	Canada	Cohort	112963	Nulliparous women with a singleton gestation who gave birth between 20–42 weeks	Not reported	Fair
2022 [89]	Italy	Cohort	126839	Nulliparous women with age at delivery between 15 and 55, gestational age between 22 and 42 weeks, and at least 4 years of traceability in healthcare databases before pregnancy	Records that lacked important information about the mother or child, as well as incorrect records, and deliveries that resulted in no babies born alive	Good
2022 [90]	Canada	Case-control	1878	≥18 years of age, had a singleton pregnancy and were enrolled at <18 weeks gestation	Preexisting diabetes, high blood pressure, autoimmune disorders, kidney disease, cardiovascular disease or chronic infection	Fair

Abbreviations: GA, gestational age; IUGR, intrauterine growth restriction; PPROM, preterm premature rupture of membranes; PTB, preterm birth; PTL, preterm labor; qfFN, quantitative fetal fibronectin; SD, standard deviation; sPTB, spontaneous preterm birth; UK, United Kingdom; USA, United States of America.
